# Time Spent on Social Media Applications in Relation to Depressive Symptoms During Emerging Adulthood and the Mediating Role of Sleep Quality: Cross-Sectional Observational Study

**DOI:** 10.2196/75337

**Published:** 2025-12-19

**Authors:** Qi Zou, Jiabi Qin, Lei Zhang, Wenhua Wang, Xiaoxiao Yuan, Tingting Wang, Yuan Peng, Ye Chen, Linfei Dou, Xinghua Yang, Xirennayi Abudurexiti, Mingyang Wu

**Affiliations:** 1 Department of Epidemiology and Health Statistics, Xiangya School of Public Health, Central South University, Changsha, China; 2 Department of Maternal, Child and Adolescent Health, School of Public Health, Kunming Medical University, Kunming, China; 3 Shaanxi Medical Association, Xi'an, Shaanxi, China; 4 Shaanxi Provincial Health Industry Association Service Center, Xi'an, Shaanxi, China; 5 Department of Maternal and Child Health, Xiangya School of Public Health, Central South University, 172 Tongzipo Road, Changsha, 410013, China, 86 15971471776

**Keywords:** emerging adulthood, social media use, depressive symptoms, sleep, mediating effect

## Abstract

**Background:**

The link between social media use and depressive symptoms remains bidirectional. Findings in this area are often compromised by methodological limitations related to measurement and sample size. As a result, it is challenging to assess dose-response relationships and potential causal pathways.

**Objective:**

This study aims to use objective measurement methods to assess the dose-response relationship and potential mechanisms between social media use and depressive symptoms.

**Methods:**

This study was conducted in 6 universities in 2022. Social media use duration was assessed based on the monitoring of mobile phone systems, and depressive symptoms were evaluated by the Self-Rating Depression Scale. Logistic regression and restricted cubic spline were used to assess the relationship between social media use and depressive symptoms. Mediation analysis was used to elucidate the biological pathways of sleep quality in the abovementioned relationship.

**Results:**

A total of 7401 college students were included in the final analysis, with 4.93% of moderate to severe depressive symptoms. After adjusting for variables such as sociodemographic characteristics and health-related characteristics, there was a significant association between individuals with longer weekly social media use time and depressive symptoms (odds ratio [OR_>48h_] 1.769, 95% CI 1.303‐2.400). Similarly, the association between instant messaging-based social media use duration and depressive symptoms was also significant (OR_>24h_ 1.728, 95% CI 1.225‐2.437), while no associations were observed for content-based social use (OR_>24h_ 1.251, 95% CI 0.932‐1.680). Restricted cubic splines regression demonstrated a J-type relationship between social media use duration and depressive symptoms. Additionally, sleep quality played a partial mediating role in the relationship between social media use duration and depressive symptoms, with the mediating effect values ranging from 24.10% to 25.25%.

**Conclusions:**

Prolonged social media use duration might be associated with an increased prevalence of depressive symptoms in emerging adulthood and may increase the odds of depression by affecting sleep quality, suggesting that early prevention and intervention regarding social media use might help to ameliorate depressive symptoms.

## Introduction

Emerging adulthood, aged 18 to 25 years, is a critical developmental period marked by identity exploration, social transition, and heightened vulnerability to environmental stressors [1,2]. During this stage, individuals navigate critical psychosocial tasks including intimate relationship development, social skill acquisition, and family role redefinition [1,2]. However, such dynamic transformations are often accompanied by heightened stressors, such as academic demands, employment competition, and shifts in living environments, which may exacerbate vulnerability to mental health disorders [3,4]. Notably, depression has emerged as a major public health issue within the college student population, manifesting through diminished motivation and cognitive fatigue [5]. A meta-analysis of Chinese cohorts reveals alarming prevalence rates, with 28.4 % of college students exhibiting depressive symptoms, significantly surpassing general population estimates [6]. These symptoms not only impair academic functioning and interpersonal communication but may escalate to severe outcomes including school withdrawal and suicidal behaviors [6,7]. Given its peak incidence during this transitional phase and substantial public health burden, there is an urgent need to investigate its underlying determinants.

In the current digital society, which was further shaped and intensified by the global COVID-19 pandemic, the daily use of social media applications has become an integral part of college students’ lives. The pandemic and associated public health measures have been widely documented to exert profound effects on the mental well-being of college students globally, including increased rates of depression, anxiety, and sleep disturbances [8-10]. This unique historical context has positioned digital platforms to play an amplified yet complex role in young adults’ lives. Although it provides college students with platforms for self-expression, information acquisition, and the establishment of social connections [11], excessive use of social media may increase the risk of emotional distress [12].

Several studies have examined the association between social media use and depression in adolescents; however, their findings remain bidirectional. A longitudinal and adolescent-based study conducted in the United States, using a combination of family-based interviews and adolescents' self-reported social media use, reported that adolescents who used social media platforms for more than 3 hours per day were more likely to have internalizing symptoms a year postbaseline follow-up [13]. Similarly, in Brazil, a study employing 3161 college students collecting data through self-administered questionnaires demonstrated that 69% reported moderate-to-severe depressive symptoms. Furthermore, the study revealed a direct relationship between the duration of social media use and depressive symptoms [14].

Conversely, studies by Alsunni and Latif [15] about college students in Saudi Arabia demonstrated that the frequency of social media use (daytime and night-time) was not associated with anxiety or depression. Aligning with these null findings, Faranda and Roberts’s study of Facebook users aged 18 to 25 years [16] also found no significant correlation between Facebook use and depressive symptoms. These inconsistencies may be attributed to regional, sociodemographic, and methodological differences. In addition, key limitations of existing studies include: (1) insufficient statistical robustness due to small sample sizes, (2) reliance on self-reported social media use through questionnaire scales, and (3) inadequate consideration of diverse social media platform impacts. To our knowledge, only 1 study has used objective measurement of social media use, focusing on 3 commonly used platforms (Facebook, Instagram, and WhatsApp). However, its small sample size (n=15) underscores the pressing need for large-scale, multicenter studies to elucidate the underlying mechanisms [17].

Prolonged exposure to electronic devices may disrupt circadian rhythms through various biological pathways, such as interfering with the function of the suprachiasmatic nucleus, the hypothalamic-pituitary-adrenal axis, and the dysregulation of melatonin secretion [18-20], thereby affecting sleep quality. Sleep problems such as insufficient sleep and difficulty falling asleep may increase an individual’s risk for symptoms such as nonsuicidal self-injury, suicidal behavior, depression, and anxiety [21-23]. Therefore, it is reasonable to hypothesize that sleep quality might play a mediating role in the association between social media use and depressive symptoms. To the best of our knowledge, some studies have investigated the mediating role of sleep quality in this context, but reliance on self-reported social media use data might result in misclassification and recall bias [24,25]. Thus, further research using systematically monitored social media data is necessary to address this issue.

Therefore, we conducted this study in 6 universities in Shaanxi Province, China. Data on the duration of social media use were collected by identifying screenshots of app activity monitored by mobile phone systems. This study aims to explore the correlation between the total use duration of social media as well as the duration of use on different platform types and depressive symptoms, as well as the underlying role of sleep quality.

## Methods

### Study Design and Participants

This cross-sectional study recruited undergraduate students from 6 universities in Shaanxi Province using a multistage random cluster sampling method during the period from October 2022 to November 2022. First, 4 out of 34 public universities and 2 out of 23 private universities in Shaanxi Province were randomly selected. Second, 2 to 4 classes were randomly sampled from all the colleges and grades of each selected university, and all the undergraduate students in the selected classes were recruited for the survey. In this study, students who refused to participate were excluded. To ensure data quality, submitted questionnaires were also excluded based on the following criteria: (1) an unreasonably short completion time (less than 500 s), which suggests random clicking; or (2) failure to correctly answer embedded logic-check questions. For example, one such question was: “Which of the following is not an animal? (A) Monkey, (B) Cow, (C) Rose, (D) Panda.” Participants selecting an option other than (C) were considered inattentive, and their data were removed. A total of 18,723 valid questionnaires were obtained. Subsequently, student questionnaires with incorrect screenshot uploads or those with an upload duration of less than 7 days were further eliminated. Ultimately, 7401 students were included in the final analysis.

The inclusion criteria were: (1) university students who agreed to participate and provided electronic informed consent, (2) individuals without major health problems or a history of mental disorders, (3) students who had not taken a leave of absence or withdrawn from school during the academic year, (4) participants who had not been involved in other similar research studies recently, and (5) students without prolonged absence for exchange programs or internships.

The exclusion criteria were: (1) nonfull-time university students, (2) individuals currently receiving psychological treatment or taking psychotropic medications, (3) participants with diagnosed sleep disorders, and (4) individuals unable to comprehend or complete the questionnaire content.

The survey was conducted by investigators who had received standardized training, and they explained the structured questionnaire to the students. A 2-stage training was carried out prior to the survey to inform the students of the purpose and process of the survey. First, 2 class cadres from each selected class and their school leaders undertook standardized training. Then, the class cadres trained all the students in each selected class and guided other classmates to fill in the structured questionnaire using quick response codes. All participants signed electronic informed consent forms.

### Ethical Considerations

This study was approved by the institutional review board of the Ethics Committee of the Second Affiliated Hospital of Xi’an Jiaotong University (approval: 2022‐248). All participants provided written electronic informed consent prior to data collection. This study was conducted in accordance with the Declaration of Helsinki and was approved by an ethics committee. To ensure privacy and confidentiality, all data were deidentified. The screenshot-based social media monitoring extracted only aggregated usage duration without accessing personal content. No compensation was provided to participants, and the manuscript contains no identifiable individual information.

### Sociodemographic Characteristics

Sociodemographic characteristics encompass gender, grade, race, area of residence, siblings, family type, and parental educational attainment.

### Social Media Use

Under the existing system, the use of social media can be obtained by screenshot recognition through “Screen Time/Healthy Mobile Phone Usage/Application Management Time” in the mobile phone system settings. Social media can be further divided into 2 major categories, namely instant messaging–based social media and content-based social media, according to their functions and positioning. Instant messaging–based social media applications encompass WeChat, QQ, and DingTalk. Content-based social media applications include Weibo, Douban, Zhihu, Xiaohongshu, Bilibili, TikTok, Kuaishou, Baidu Tieba, Zuiyou, miHoYo Community, NetEase LOFTER, Twitter, Today Campus, and PU Pocket Campus. Subsequently, the use durations in the past week for each category can be calculated respectively. GWI’s latest figures indicate that the typical social media user spends 2 hours and 21 minutes using social media each day [26]. Taking into account multiple factors such as university course studies and social practice, this study defines excessive social media use as exceeding 24 hours per week on social media platforms, serving as the reference group.

### Depressive Symptoms

The Self-Rating Depression Scale, developed by Zung [27], was used to measure depressive symptoms. This scale consists of 20 items and assesses 10 positive symptoms and 10 negative symptoms over the past nearly 1 week. The scoring range for all items is from 1 (none or a little of the time) to 4 (most or all of the time). The higher the score, the more severe the depressive symptoms are. A standard score higher than 50 indicates depression. For the purpose of this study, we created a binary outcome variable by combining no and mild depressive symptoms into 1 reference group, contrasted with moderate-to-severe symptoms. This approach was determined by our research focus on identifying factors associated with more substantial depressive symptomatology rather than mild mood variations [28]. In this study, the Cronbach *α* coefficient of this scale was 0.88.

### Sleep Quality

The Pittsburgh Sleep Quality Index, developed by Buysse et al [29], is a scale used to assess sleep quality in the past month. The overall Pittsburgh Sleep Quality Index score generated by summarizing the total scores of 7 factors ranges from 0 to 21, and a score of 8 or higher is defined as a sleep disorder. Among them, a sleep latency higher than 30 minutes is defined as prolonged sleep latency [30], and a sleep time of less than 7 hours at night is defined as insufficient sleep [31]. In this study, the Cronbach *α* coefficient of this scale was 0.85.

### Health-Related Characteristics

We also collected information on health-related characteristics, including smoking, drinking, height and weight, and physical activity. Smoking was defined as actively smoking one or more cigarettes within the past 30 days. Drinking was defined as having consumed at least 1 type of alcoholic beverage with an alcohol content exceeding 10 grams within the past 30 days. BMI was calculated by dividing weight (kg) by the square of height (m). The physical activity was measured using the International Physical Activity Questionnaire-Short Form, which captures physical activity across multiple domains: occupational activities, transportation, household and gardening tasks, and leisure-time activities (including sports and exercise). According to the standard cut-off levels for calculating metabolic equivalents, physical activity categories were divided into low, moderate, and high levels [32].

### Statistical Analysis

In this study, SPSS (version 25.0; IBM Corp) and R (version 4.4.2; R Foundation for Statistical Computing) software were used for statistical analysis. For categorical data, frequency-based indicators were adopted for statistical description, while for quantitative data, the description was carried out by means of mean (SD) or median and IQR. Chi-square test or 2-tailed *t* test was used to compare the distribution of different characteristics. For covariates with missing values constituting less than 5%, simple imputation methods were applied based on the distributional characteristics of the covariates. For continuous variables, missing values were imputed by random sampling from a normal distribution with mean and SD derived from the observed data. For categorical variables, missing values were imputed by random sampling from a uniform distribution based on the probability distribution of the observed categories. Had any covariate contained missing values exceeding the 5 % threshold, multiple imputation would have been used.

Three sets of binary logistic regression models were constructed for each association (social media use and depressive symptoms, social media use and sleep quality, and sleep quality and depressive symptoms) to estimate the odds ratio (OR) and 95% CI. Model 1 was adjusted based on gender and grade. Model 2 further incorporated the area of residence, siblings, and parental educational attainment as control covariates. Model 3 continued to adjust for BMI and physical activity. Subgroup analyses were conducted based on fully adjusted multivariate models to examine the association between social media use and depressive symptoms, with stratification by grade, gender, race, area of residence, and siblings.

To flexibly model the dose-response relationship between social media use duration (a continuous variable) and depressive symptoms, we used a restricted cubic spline, which was chosen for its ability to uncover potential nonlinear patterns without strong prior assumptions. Finally, a mediation effect model was used to analyze the mediating role of sleep quality in the impact of social media use on depressive symptoms among college students. The key assumptions include no unmeasured confounding in the relationships between (1) social media use and sleep, (2) social media use and depression, and (3) sleep and depression. A significance level of *α*=.05 was set, with *P*<.05 indicating statistical significance.

## Results

### Baseline Characteristics of Participants

Table 1 presents the basic characteristics of the participants. Among the 7401 participants, the detection rate of sleep disorders was 16.94% (n=1254), and the detection rate of moderate to severe depressive symptoms was 4.93% (n=365). Overall, the proportion of women was relatively high (n=5017, 67.79%). The majority of students were from families with biological parents (n=6500, 87.83%). More than 70% of the students had siblings (n=5259, 71.06%). The educational level of their parents was mainly junior high school or below. Students with smoking (n=785, 10.61%) or drinking (n=1466, 19.81%) experiences accounted for a minority, and 73.02% (n=5404) of the students exhibited a low level of physical activity in daily life.

**Table 1.** Baseline characteristics of participants.

Characteristics	Overall (n=7401), n (%)	Sleep disorders (n=1254), n (%)	Depressive symptoms (n=365), n (%)
Grade			
1st	2199 (29.71)	299 (23.84)	92 (25.2)
2nd	1618 (21.86)	288 (22.97)	94 (25.8)
3rd	1797 (24.28)	343 (27.35)	108 (29.6)
4th+	1787 (24.15)	324 (25.84)	71 (19.5)
Gender (man)	2384 (32.21)	372 (29.67)	121 (33.2)
Race (Han)	7200 (97.28)	1211 (96.57)	347 (95.1)
BMI			
Underweight	1443 (19.50)	266 (21.21)	77 (21.1)
Normal weight	4473 (60.44)	741 (59.09)	213 (58.4)
Overweight	919 (12.42)	140 (11.16)	48 (13.2)
Obesity	566 (7.65)	107 (8.53)	27 (7.4)
Area of residence (urban)	3474 (46.94)	594 (47.37)	176 (48.2)
Family type^a^			
Dual-parent family type 1	6500 (87.83)	1058 (84.37)	305 (83.6)
Dual-parent family type 2	300 (4.05)	54 (4.31)	15 (4.1)
Single-parent family	558 (7.54)	128 (10.21)	38 (10.4)
Others	43 (0.58)	14 (1.12)	7 (1.9)
Siblings (no)	2142 (28.94)	361 (28.79)	109 (29.9)
Maternal educational attainment			
Middle school or under	4804 (64.91)	829 (66.11)	225 (61.6)
High school	1535 (20.74)	251 (20.02)	70 (19.2)
College or above	1062 (14.35)	174 (13.88)	70 (19.2)
Parental educational attainment			
Middle school or under	4141 (55.95)	735 (58.61)	208 (57.0)
High school	1647 (22.25)	267 (21.29)	80 (21.9)
College or above	1613 (21.79)	252 (20.10)	77 (21.1)
Smoking (yes)	785 (10.61)	209 (16.67)	86 (23.6)
Drinking (yes)	1466 (19.81)	367 (29.27)	118 (32.3)
Physical activity			
Low	5404 (73.02)	961 (76.63)	291 (79.7)
Moderate	1363 (18.42)	187 (14.91)	43 (11.8)
High	634 (8.57)	106 (8.45)	31 (8.5)

a Dual-parent family type 1 refers to a family where both parents are biological; dual-parent family type 2 refers to a dual-parent family consisting of 1 biological parent and 1 step-parent.

### Associations of Social Media Use With Depressive Symptoms

The relationship between social media use and depressive symptoms is shown in Table 2. Overall, there was a positive association between the increase in the duration of social media use and the elevation of the odds of depression. After adjusting for gender and grade (model 1 in Table 2), it could be observed that as the duration of social media use extends, the likelihood of depression increased accordingly (*P* value for trend <.001). After further taking into account race, area of residence, siblings, maternal educational attainment, and paternal educational attainment (model 2 in Table 2), the results remained consistent. In the model that continues to incorporate BMI and physical activity (model 3 in Table 2), although the ORs declined slightly (OR_>48h_ 1.769, 95% CI 1.303‐2.400), the positive correlation between social media use and depression still existed. Regarding the association between the use of instant messaging–based social media and depression, the results were consistent with the overall use situation (OR_>24h_ 1.728, 95% CI 1.225‐2.437); however, this was not the case for the use of content-based social media and depression (OR_>24h_ 1.251, 95% CI 0.932‐1.680). The subgroup analysis results demonstrated relative stability for overall social media use and instant messaging–based social media; however, the association between content-based social media and depression remained statistically nonsignificant (Figure S1 in Multimedia Appendix 1).

**Table 2.** Associations of social media use with depressive symptoms.

Variables	Model 1^a^, OR^b^ (95% CI)	Model 2^c^, OR (95% CI)	Model 3^d^, OR (95% CI)
Social media use (h)	1.101 (1.055-1.148)^e^^f^	1.099 (1.054-1.147)^e^^f^	1.097 (1.052-1.144)^e^^f^
≤24	Ref	Ref	Ref
24-36	1.058 (0.760-1.472)	1.067 (0.766-1.486)	1.069 (0.767-1.489)
36-48	1.377 (1.012-1.873)^f^	1.389 (1.020-1.891)^f^	1.382 (1.015-1.883)^f^
>48	1.777 (1.310-2.409)^f^	1.784 (1.315-2.421)^f^	1.769 (1.303-2.400)^f^
*P* value for trend	<.001	<.001	<.001
Instant messaging–based social media (h)	1.138 (1.071-1.208)^e^^f^	1.136 (1.070-1.207)^e^^f^	1.137 (1.070-1.207)^e^^f^
≤7	Ref	Ref	Ref
7-16	0.954 (0.664-1.369)	0.950 (0.662-1.365)	0.945 (0.658-1.358)
16-24	1.392 (0.975-1.987)	1.391 (0.974-1.987)	1.401 (0.981-2.002)
>24	1.738 (1.234-2.448)^f^	1.721 (1.222-2.426)^f^	1.728 (1.225-2.437)^f^
*P* value for trend	<.001	<.001	<.001
Content–based social media (h)	1.076 (1.016-1.139)^e^^f^	1.076 (1.016-1.140)^e^^f^	1.072 (1.012-1.135)^e^^f^
≤7	Ref	Ref	Ref
7-16	0.979 (0.715-1.342)	0.978 (0.713-1.341)	0.971 (0.708-1.331)
16-24	1.088 (0.798-1.484)	1.088 (0.797-1.485)	1.083 (0.793-1.478)
>24	1.273 (0.949-1.708)	1.273 (0.948-1.708)	1.251 (0.932-1.680)
*P* value for trend	.07	.07	.09

a Model 1: adjustment for gender and grade.

b OR: odds ratio.

c Model 2: adjustment for gender, grade, race, area of residence, siblings, maternal educational attainment, paternal educational attainment.

d Model 3: adjustment for gender, grade, race, area of residence, siblings, maternal educational attainment, paternal educational attainment, BMI, and physical activity.

e ORs represent the effect size of a 7-hour weekly increase in exposure time.

f *P<.05.

### Dose-Response Relationship Between Social Media Use and Depressive Symptoms

**Figure 1.**
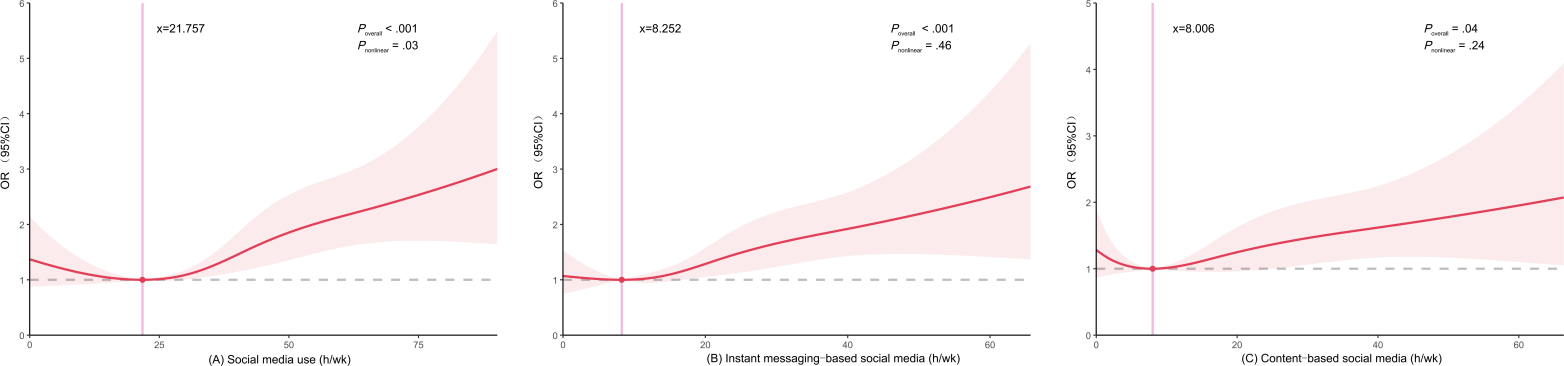
(A) The dose-response relationships of overall social media use, (B) instant messaging–based social media, and (C) content-based social media with depressive symptoms. The analysis was adjusted for gender, grade, race, area of residence, siblings, maternal educational attainment, paternal educational attainment, BMI, and physical activity. OR: odds ratio.

Restricted cubic spline regression showed that the association between the overall use of social media and depressive symptoms presented a J-shaped curve (*P-*nonlinear <.001; Figure 1A). Although both instant messaging–based social media and content-based social media exhibited an approximately J-shaped relationship with depressive symptoms, statistical inference did not support the existence of a nonlinear relationship. Specifically, as the duration of use of these 2 types of social media increased, the likelihood of depressive symptoms showed a monotonically increasing trend (*P-*nonlinear >.05; Figure 1B and C).

The relationship between social media use and sleep quality is presented in Table S1 in Multimedia Appendix 1. Whether it was the overall use of social media (OR_>48h_ 1.493, 95% CI 1.298‐1.717), or the use of instant messaging–based social media (OR_16~24h_ 1.196, 95% CI 1.027‐1.393) or content-based social media (OR_>24h_ 1.630, 95% CI 1.423‐1.868), all of them might lead to the prolongation of sleep latency. In addition, excessive use of social media would increase the likelihood of sleep disorders (OR_>48h_ 1.357, 95% CI 1.142‐1.611). Multiple indicators of sleep quality (prolonged sleep latency OR 2.313, 95% CI 1.783‐3.001; insufficient sleep OR 2.073, 95% CI 1.668‐2.576; sleep disorders OR 7.782, 95% CI 6.245‐9.697) were all associated with depression (Table S2 in Multimedia Appendix 1).

### Mediating Effect Analysis

**Figure 2.**
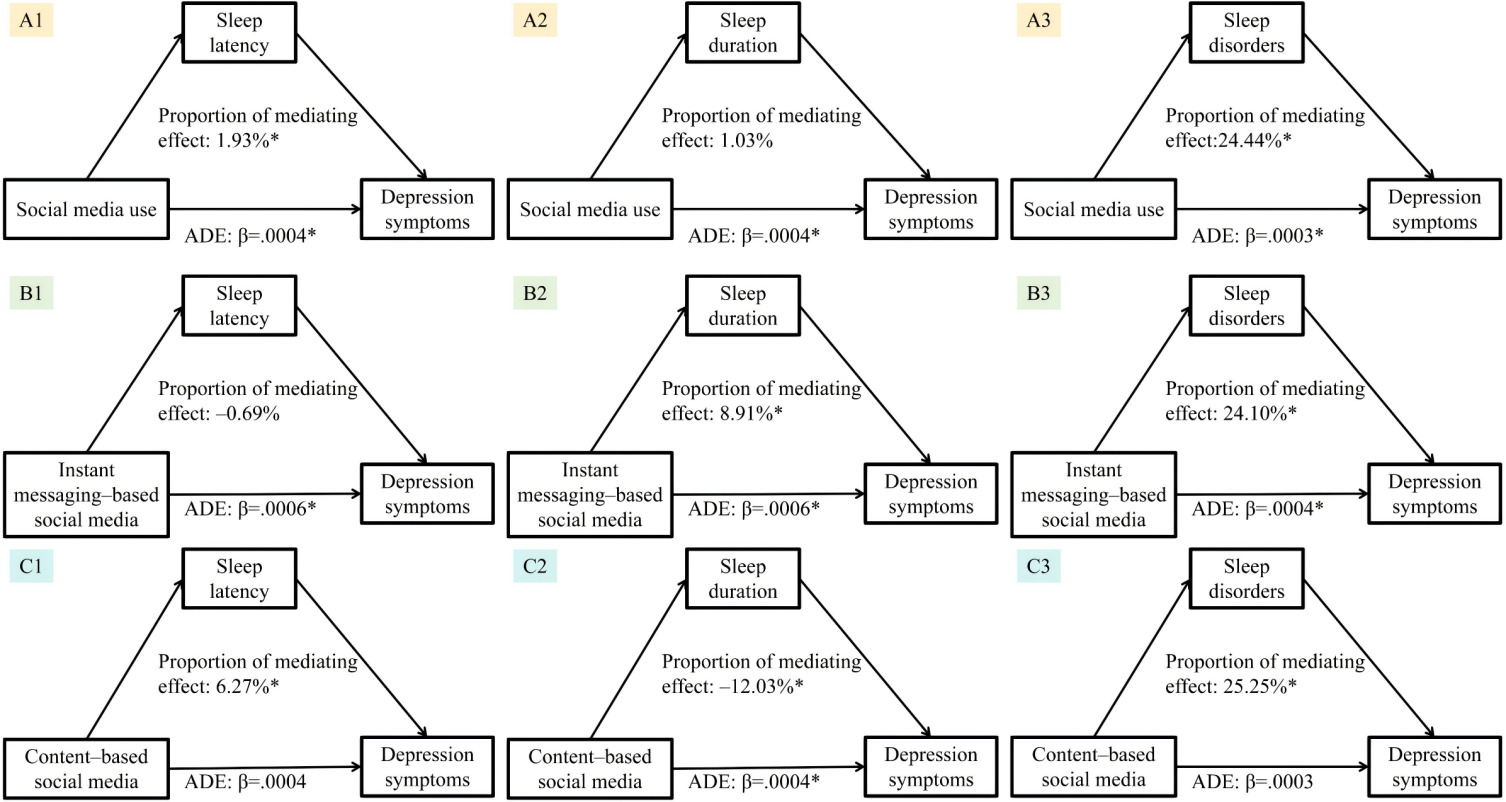
The mediation effect of sleep quality (sleep latency, sleep duration, and sleep disorders) on the association between social media use (social media use, instant messaging–based social media, and content-based social media) and depressive symptoms. Percentages shown indicate the proportion of the total effect mediated through sleep quality (*P<*.05). The analysis was adjusted for gender, grade, race, area of residence, siblings, maternal educational attainment, paternal educational attainment, BMI, and physical activity. Negative percentages for the proportion mediated occur in an inconsistent mediation model, where the direct and indirect effects operate in opposite directions. This indicates that, while the overall relationship between social media use and depressive symptoms is positive, the mediation through sleep quality reveals a more complex mechanism: the negative direct effect suggests potential protective aspects of social media use, which are masked by its strong indirect detrimental effect via impaired sleep. ADE: average direct effect.

Figure 2 shows the results of the mediating effect analysis of multiple sleep quality indicators in the association between social media use and depressive symptoms. Sleep disorders played a partial mediating role in the association between social media use (including instant messaging–based social media and content-based social media) and depressive symptoms, with the mediating effect values being 24.44%, 24.10%, and 25.25% respectively (Figure 2 A3, B3, C3).

## Discussion

### Principal Findings

This study characterized the association between social media use and depressive symptoms in emerging adulthood as being dose-dependent, partially mediated by sleep quality, and varying by platform type. Specifically, our analyses demonstrated that there is a significantly positive correlation between the increased duration of social media use among college students and the rising prevalence of depressive symptoms. Even after adjusting for multiple covariates, this association remains stable. The link between the use of instant messaging–based social media and depressive symptoms is consistent with that of overall use of social media, whereas this is not observed for content-based social media. Restricted cubic spline regression analysis revealed a J-shaped relationship between the overall social media use and depressive symptoms. Moreover, sleep disorders play a partial mediating role between social media use and depressive symptoms, with the mediating effect ranging from 24.10% to 25.25%.

In this study, a significant association between social media use and depressive symptoms was found. This result is consistent with previous studies [33-35]. Yan et al [33] surveyed 568 college students using a brief scale to assess depressive emotions and mobile social media use, finding a positive correlation between the intensity of mobile social media use and depressive emotions. Additionally, Lin et al [34] investigated the social media use and depression among 1787 adults aged between 19 and 32 years and concluded that social media use was significantly associated with an increased risk of depression, with a strong linear dose-response trend. However, it is worth noting that some studies conducted among the older population have suggested that engaging in social media activities is not only associated with a lower risk of depressive symptoms in nondepressed individuals, but also promotes the psychosocial health level of middle-aged and older people [36,37]. This is contrary to our findings that the use of social media is associated with depression among young people and adolescents [38,39]. The possible explanations underlying the diverse results from different studies might be attributed to variations in exposure assessment methodologies, differential population susceptibility, and discrepancies in sample sizes. Most prior studies have relied on self-reported questionnaires to assess the duration of social media or smartphone use—an approach constrained by recall bias, social desirability bias, and limited temporal precision. In contrast, our study used systematically monitored smartphone use data to quantify both total social media engagement duration and platform-specific usage patterns, thereby circumventing self-reporting biases and yielding more precise, objective behavioral metrics. Therefore, our findings extend the limited available evidence on the association of social media use and its subtypes with the increased prevalence of depressive symptoms among emerging adults, while also providing robust data to reveal the dose-response relationship between the two.

The results of the study demonstrated that social media use could influence the depressive mood of college students through the partial mediating effect of sleep quality, which was consistent with the findings of previous studies [40]. Excessive use of electronic devices such as mobile phones can increase exposure to blue light, suppress the secretion of melatonin, and keep the brain in an active thinking state [41,42]. Or it might reduce or postpone bedtime due to waking up to check for updates on social media apps and receive or send messages [43,44]. And social comparison and information overload on social media might also trigger anxiety and stress [33]. All of the abovementioned pathways are likely to decrease the sleep quality of individuals. Based on previous studies, it is not difficult to find that lack of sleep may impair the function of the prefrontal cortex, leading to abnormal emotional regulation function [45]. Or it may increase the expression of inflammatory markers by activating the sympathetic nervous system and β-adrenergic signaling, thereby activating the inflammatory response and increasing the risk of depression [46,47]. According to the learned helplessness model, if individuals continuously experience sleep problems, they will think that they are unable to solve the problems, thus generating negative emotions, and over time, a vicious cycle will be formed [45].

The findings of this study revealed a J-shaped association between total weekly social media engagement duration and depressive symptoms among college students, aligning with previous research findings [48]. Specifically, individuals who either excessively or minimally engage with social media exhibit a higher propensity for depression, whereas moderate use appears to be associated with a lower risk. This potential protective effect may stem from the opportunities social media provides for self-expression, information acquisition, and the establishment of social connections, which can enhance self-esteem, increase social capital, and foster social support, thereby mitigating the impact of negative emotions [11,43]. Notably, although both instant messaging–based social media and content-based social media platforms demonstrated a similar J-shaped curve in their association with depressive symptoms, multivariate-adjusted models ultimately revealed a linear dose-response relationship.

The J-shaped curve observed between overall social media use and depressive symptoms carries important implications. It suggests that the relationship is not one of simple linear harm, but rather that moderate use may be associated with the lowest risk, potentially even conferring some benefits related to social connection and support. Conversely, the sharply elevated risk at both the lowest and highest ends of use indicates that both abstinence and excessive immersion in the digital social environment may be detrimental to mental well-being. This nonlinear pattern underscores the limitation of categorizing social media use as merely “good” or “bad,” and instead calls for a more nuanced public health message that focuses on finding an optimal balance. Importantly, the impact of social media use on depression may not only depend on the duration of use but also on the specific types of social media activities [36], underscoring the need for future research to move beyond mere duration metrics and explore the nuanced relationships between users’ social media behaviors and mental health outcomes.

The association between prolonged social media use and poor mental health outcomes underscores a critical public health issue for emerging adults. Our results highlight the potential benefits of promoting mindful social media use and improving sleep hygiene as preventative strategies. Furthermore, our findings add to the scientific foundation that is currently informing a global conversation, including regulatory discussions in some countries regarding age-specific social media access. Ultimately, protecting the mental well-being of young people in the digital era will require a multifaceted approach, combining individual-level interventions, parental education, and evidence-based policy-making.

### Strengths and Limitations

This study was conducted in late 2022, a time when universities in China had resumed normal in-person teaching and campus activities, albeit within the broader context of the postpandemic era. It is possible that the lingering effects of the COVID-19 pandemic could have influenced the baseline prevalence of depressive symptoms and sleep patterns in the general student population. However, the primary aim of our research was not to estimate the absolute prevalence of these conditions, but to examine the relationships between social media use behaviors and mental health outcomes. The dose-response relationships and the mediating role of sleep quality identified in our analysis represent intrinsic associations that are grounded in established biological and psychological mechanisms. Therefore, we have reason to believe that these core findings about the interplay between digital behavior and mental well-being are robust and extend their relevance to the emerging adulthood population in the current era of ubiquitous digital connectivity.

Our study possesses notable strengths. First, it is a large-scale survey targeting the college student population. With a substantial sample size and relatively strong representativeness, the research findings possess high credibility and are of great value for generalization. Second, the overall use of social media and the duration of using different functional types of social media among college students were precisely measured by means of monitoring through mobile phone systems. In contrast to the traditional self-reporting method for duration, this approach can offer more precise and objective data, minimizing the potential biases that may exist in users’ self-reports to the greatest extent possible.

This study also has certain limitations. First, the cross-sectional study design restricts the causal inferences of this study. Future studies could also consider adopting longitudinal designs or experimental studies to more accurately assess the causal impact of social media use on depression. Second, this study did not specifically assess social media usage duration during particular time frames (eg, nighttime) or evaluate potentially mood-influencing social media activities (such as chatting, reading news, and watching video), thereby precluding a clear analysis of the association between such behavioral patterns and depressive symptoms. Third, the directionality in the mediation model, while theoretically grounded, cannot be conclusively established due to the potential for bidirectional relationships between sleep quality and depressive symptoms. Future research should use longitudinal designs to verify the temporal sequence of these associations. Fourth, this study may be subject to potential selection bias due to the substantial proportion of participants who were excluded from the final analysis primarily because of incomplete or invalid screenshot data on social media use. This potential bias may limit the generalizability of our findings to the broader college student population. Future studies should develop more user-friendly and robust methods for collecting objective digital behavioral data to minimize such exclusions.

### Conclusions

Excessive social media use elevates the likelihood of depressive symptoms among college students. The J-shaped curve relationship observed between the overall duration of social media use and depressive symptoms suggests that both excessive use and complete nonuse may be associated with higher risk, highlighting the importance of promoting balanced, rather than simply minimal, social media engagement as a public health goal. Moreover, sleep disorder exerts a partial mediating effect between social media use and depressive symptoms.

## Supplementary material

10.2196/75337 Multimedia Appendix 1 Subgroup analyses and statistical tables regarding the associations between social media use, sleep quality, and depressive symptoms.

## Abbreviations

OR: odds ratio
