# Supplementary material for: Time Spent on Social Media Applications in Relation to Depressive Symptoms During Emerging Adulthood and the Mediating Role of Sleep Quality: Cross-Sectional Observational Study
Source: J Med Internet Res. 2025 Dec 19;27:e75337. doi: 10.2196/75337 (PMC12716832; doi:10.2196/75337)
Supplement: Multimedia Appendix 1 [file jmir-v27-e75337-s001.pdf]

Supplementary material

Supplementary Figure

Figure S1. The subgroup analysis of overall social media use (A), instant messaging-based social media (B), and content-based social media (C) with depressive symptoms.

Supplementary Tables

Table S1. Associations of Social Media Use with Sleep quality.

Table S2. Associations of Sleep quality with Depressive symptoms.

Figure S1. The subgroup analysis of overall social media use (A), instant messaging-based social media (B), and content-based social media (C) with depressive symptoms.

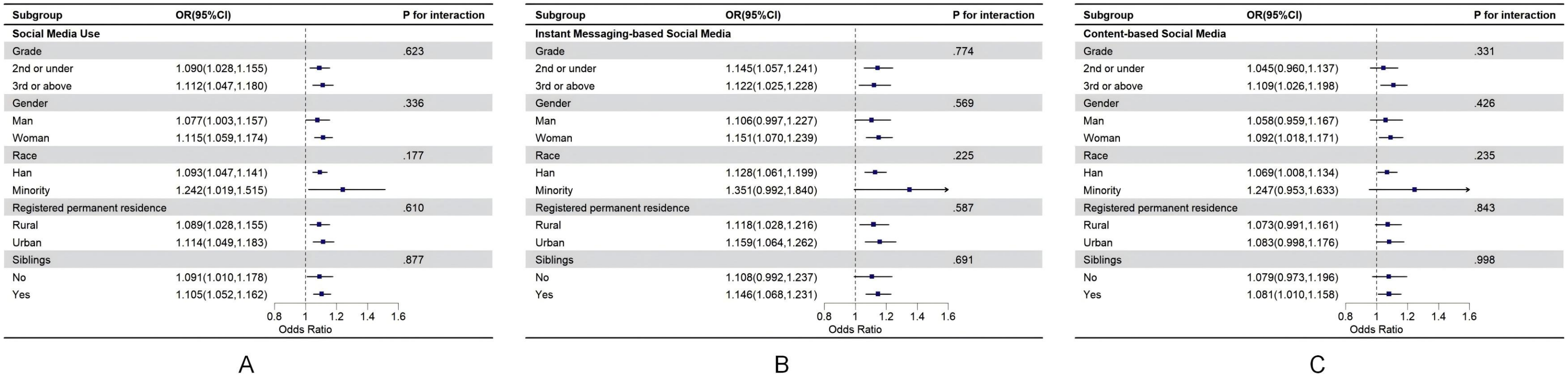

Note: *OR*s represent the effect size of a 7-hour weekly increase in exposure time. *OR*, odds ratio; *CI,* confidence interval. The analysis was adjusted for gender, grade, race, area of residence, siblings, maternal educational attainment, paternal educational attainment, BMI, and physical activity.

Table S1 Associations of Social Media Use with Sleep quality.

Variables	Model 1	Model 2	Model 3
	***OR*(95%*CI*)**	***OR*(95%*CI*)**	***OR*(95%*CI*)**
Prolonged sleep latency			
Social Media Use			
≤24h	Ref	Ref	Ref
24~36h	1.062(0.930,1.213)	1.071(0.937,1.225)	1.075(0.940,1.229)
36~48h	1.172(1.028,1.336)*	1.176(1.031,1.341)*	1.176(1.031,1.342)*
>48h	1.463(1.273,1.680)*	1.491(1.296,1.714)*	1.493(1.298,1.717)*
P value for trend	<.001	<.001	<.001
Instant Messaging-based Social Media			
≤7h	Ref	Ref	Ref
7~16h	1.090(0.944,1.259)	1.098(0.950,1.270)	1.099(0.951,1.271)
16~24h	1.180(1.014,1.373)*	1.189(1.021,1.384)*	1.196(1.027,1.393)*
>24h	1.038(0.895,1.203)	1.050(0.905,1.218)	1.057(0.911,1.227)
P value for trend	.641	.550	.475
Content-based Social Media			
≤7h	Ref	Ref	Ref
7~16h	1.059(0.928,1.209)	1.070(0.937,1.223)	1.069(0.935,1.222)
16~24h	1.336(1.166,1.531)*	1.358(1.184,1.558)*	1.362(1.187,1.562)*
>24h	1.605(1.403,1.837)*	1.640(1.431,1.878)*	1.630(1.423,1.868)*
P value for trend	<.001	<.001	<.001
Insufficient sleep			

**Variables**	**Model 1**	**Model 2**	**Model 3**
	***OR*(95%*CI*)**	***OR*(95%*CI*)**	***OR*(95%*CI*)**
Social Media Use			
≤24h	Ref	Ref	Ref
24~36h	0.859(0.741,0.994)*	0.854(0.737,0.990)*	0.855(0.737,0.990)*
36~48h	0.894(0.774,1.032)	0.887(0.768,1.024)	0.886(0.767,1.023)
>48h	0.932(0.804,1.081)	0.921(0.794,1.069)	0.920(0.793,1.067)
P value for trend	.417	.332	.323
Instant Messaging-based Social Media			
≤7h	Ref	Ref	Ref
7~16h	0.949(0.807,1.116)	0.943(0.801,1.109)	0.942(0.801,1.109)
16~24h	1.055(0.892,1.248)	1.048(0.886,1.240)	1.049(0.887,1.242)
>24h	1.316(1.118,1.548)*	1.310(1.113,1.542)*	1.311(1.114,1.543)*
P value for trend	<.001	<.001	<.001
Content-based Social Media			
≤7h	Ref	Ref	Ref
7~16h	0.917(0.794,1.059)	0.909(0.786,1.050)	0.907(0.785,1.049)
16~24h	0.865(0.747,1.002)	0.852(0.735,0.988)*	0.852(0.735,0.987)*
>24h	0.700(0.604,0.811)*	0.686(0.592,0.796)*	0.684(0.590,0.794)*
P value for trend	<.001	<.001	<.001
Sleep disorders			
Social Media Use			
≤24h	Ref	Ref	Ref
24~36h	0.961(0.805,1.147)	0.963(0.807,1.150)	0.964(0.807,1.151)

Variables	Model 1	Model 2	Model 3
	***OR*(95%*CI*)**	***OR*(95%*CI*)**	***OR*(95%*CI*)**
36~48h	1.011(0.850,1.202)	1.008(0.848,1.199)	1.005(0.845,1.195)
>48h	1.366(1.150,1.621)*	1.365(1.149,1.621)*	1.357(1.142,1.611)*
P value for trend	<.001	.001	.001
Instant Messaging-based Social Media			
≤7h	Ref	Ref	Ref
7~16h	0.972(0.801,1.178)	0.971(0.800,1.178)	0.967(0.797,1.173)
16~24h	1.083(0.887,1.322)	1.082(0.886,1.321)	1.084(0.888,1.324)
>24h	1.395(1.151,1.690)*	1.396(1.152,1.691)*	1.394(1.150,1.690)*
P value for trend	<.001	<.001	<.001
Content-based Social Media			
≤7h	Ref	Ref	Ref
7~16h	1.059(0.889,1.260)	1.060(0.891,1.262)	1.054(0.885,1.255)
16~24h	1.068(0.895,1.273)	1.062(0.890,1.267)	1.057(0.886,1.262)
>24h	1.043(0.878,1.239)	1.036(0.872,1.231)	1.026(0.863,1.220)
P value for trend	.643	.721	.792

Note: *OR*, odds ratio; *CI*, confidence interval. Model 1, adjustment for gender, grade. Model 2, adjustment for gender, grade, race, area of residence, siblings, maternal educational attainment, paternal educational attainment. Model 3, adjustment for gender, grade, race, area of residence, siblings, maternal educational attainment, paternal educational attainment, BMI, and physical activity.*$p < .05$.

Table S2 Associations of Sleep quality with Depressive symptoms.

**Variables**	**Model 1**	**Model 2**	**Model 3**
	***OR*(95%*CI*)**	***OR*(95%*CI*)**	***OR*(95%*CI*)**
Sleep latency			
<30min	Ref	Ref	
≥30min	2.275(1.757,2.946)*	2.336(1.801,3.030)*	2.313(1.783,3.001)*
Sleep duration			
≥7h	Ref	Ref	
<7h	2.082(1.677,2.585)*	2.076(1.671,2.580)*	2.073(1.668,2.576)*
Sleep disorders			
No	Ref	Ref	
Yes	7.867(6.320,9.792)*	7.875(6.321,9.810)*	7.782(6.245,9.697)*

Note: *OR*, odds ratio; *CI*, confidence interval. Model 1, adjustment for gender, grade. Model 2, adjustment for gender, grade, race, area of residence, siblings, maternal educational attainment, paternal educational attainment. Model 3, adjustment for gender, grade, race, area of residence, siblings, maternal educational attainment, paternal educational attainment, BMI, and physical activity.*$p < .05$.
